# Molecular Neuroprotective Responses to *Achyrocline satureioides* Extract in *Caenorhabditis elegans*: Modulation of Conserved FOXO/NRF2 Pathways and Resistance to Neurotoxic Insults

**DOI:** 10.1007/s12035-026-06212-0

**Published:** 2026-09-24

**Authors:** Péterson Alves Santos, Eduardo Mariano de Andrade, Ionara Rodrigues Siqueira, Patrícia Pereira

**Affiliations:** Department of Pharmacology, Institute of Basic Health Sciences, Federal University of Rio Grande Do Sul, Porto Alegre, 2600 Brazil

**Keywords:** Achyrocline satureioides, Caenorhabditis elegans, Neuroprotection, Neurotoxicity, Oxidative stress

## Abstract

*Achyrocline satureioides* is widely consumed as an herbal infusion in South America; however, the toxicological profile of its aqueous extract (ASAE), particularly under conditions of neurotoxic stress, remains incompletely characterized. In the present study, we investigated the safety profile of ASAE and its capacity to modulate chemically induced neurotoxicity by integrating in silico toxicological predictions with in vivo assays in *Caenorhabditis elegans*. Wild-type and mutant strains deficient in key stress-response regulators (*daf-2, daf-16,* and *skn-1*) were exposed to ASAE (10–50 mg/mL). Toxicological and neurofunctional endpoints included survival under methylmercury (MeHg) exposure, PTZ-induced paralysis, locomotor performance, lipofuscin accumulation as a biomarker of oxidative damage, and activation of stress-responsive signaling pathways. Chronic ASAE exposure did not induce developmental toxicity or reduce lifespan at the tested concentrations. Notably, ASAE pre-exposure was associated with reduced behavioral deficits induced by MeHg and delayed PTZ-induced paralysis, indicating reduced susceptibility to chemically induced neurotoxic insults. Mechanistic analyses suggested that these findings are consistent with the involvement of conserved stress-response pathways involving *daf-16* and *skn-1*, regulators functionally analogous to mammalian FOXO and NRF2 signaling. Consistent with these findings, computational toxicology predicted low systemic toxicity for major ASAE constituents and limited interaction with toxicity-related biomarkers. Collectively, these results indicate that ASAE exhibits a favorable toxicological profile and modulates conserved molecular pathways that enhance resilience to neurotoxic stress in *C. elegans*.

**Graphical Abstract:**

**Figure:**
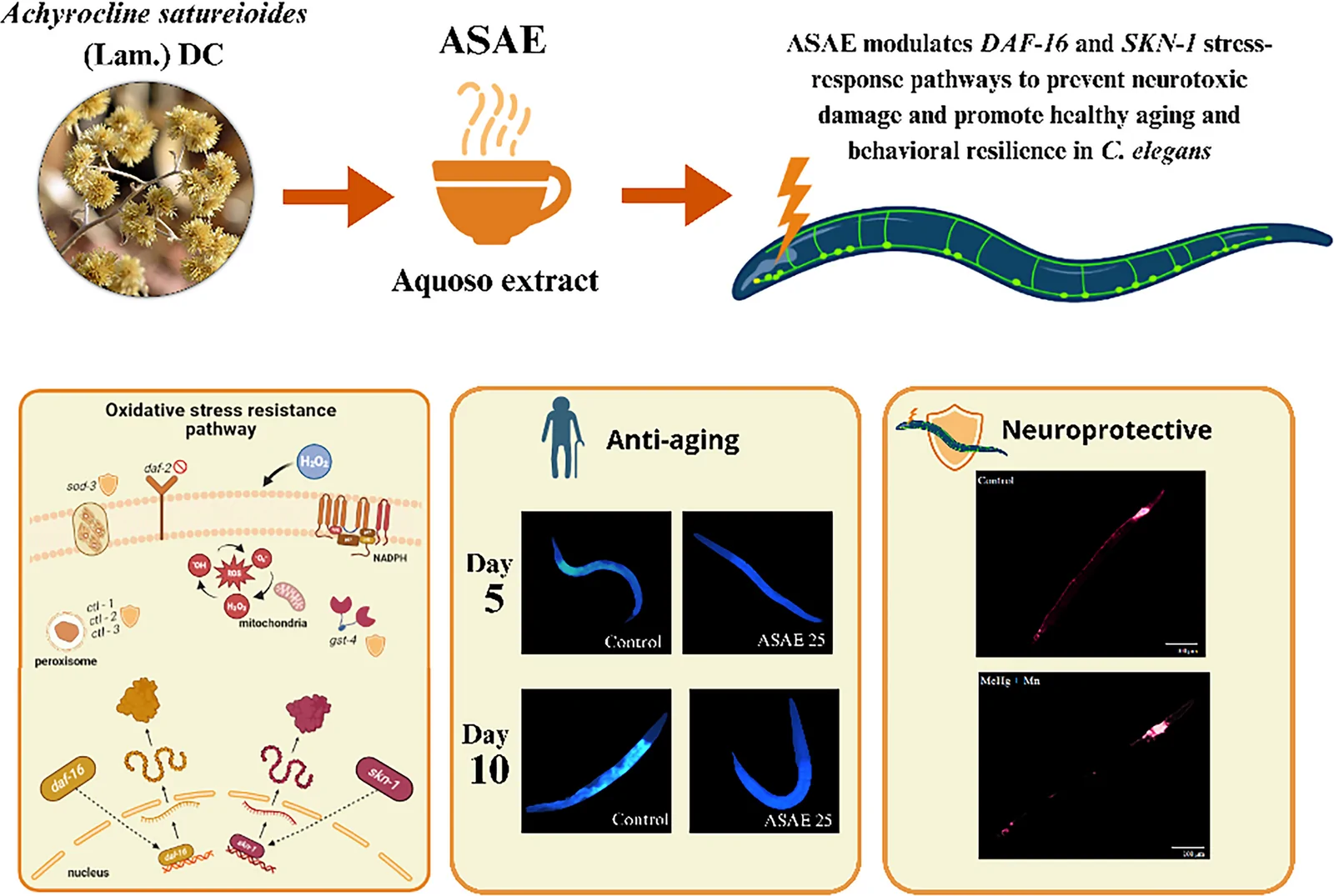

**Supplementary Information:**

The online version contains supplementary material available at https://doi.org/10.1007/s12035-026-06212-0.

## Introduction

Neuronal systems are highly vulnerable to xenobiotic-induced oxidative and excitatory stress, processes that contribute to the pathophysiology of neurodegenerative disorders. Neurotoxicity represents a critical endpoint in toxicological evaluation, as neuronal tissue is highly sensitive to redox imbalance and xenobiotic interference. Oxidative stress and disruption of detoxification pathways are central mechanisms underlying chemically induced neurotoxicity. Although antioxidant-rich extracts are frequently described as biologically protective, their interaction with conserved stress-response regulators may alter neuronal resilience depending on dose, exposure duration, and toxicant context [1].

*Achyrocline satureioides* (Lam.) DC., commonly known as “marcela,” is a South American plant widely consumed as an herbal infusion. Its extensive traditional use, particularly in southern Brazil, has led to increasing exposure of human populations to complex mixtures of bioactive secondary metabolites, including flavonoids such as quercetin, luteolin, and 3-O-methylquercetin. These compounds are recognized for their biological reactivity and capacity to interact with conserved cellular signaling pathways.

Although previous investigations indicate minimal acute and subchronic toxicity at traditional doses [2], the toxicological profile of aqueous extracts remains incompletely characterized.Recent evidence indicates that major flavonoids present in *Achyrocline satureioides*, including quercetin, luteolin, and 3-O-methylquercetin, are capable of modulating oxidative stress, neuroinflammatory responses, mitochondrial homeostasis, and conserved neuronal signaling pathways associated with cellular resilience [3, 4]. Several of these compounds have also demonstrated neuroactive properties and the ability to interact with central nervous system pathways in experimental models [5]. In addition, previous studies have suggested protective effects of *A. satureioides* and its isolated constituents in neuronal and oxidative stress-related contexts, including protection against rotenone-induced toxicity in human neural cells [6, 7]. Based on these observations, the present study focused specifically on neurotoxicity-related endpoints and conserved stress-response pathways in *C. elegans*. Other systemic toxicological endpoints were not prioritized because the primary objective was to investigate the interaction between ASAE and chemically induced neuronal stress conditions, particularly those associated with oxidative imbalance and neurofunctional impairment.

The nematode *Caenorhabditis elegans* provides a well-established in vivo model for toxicological pathway analysis, enabling the investigation of conserved stress and detoxification mechanisms. In this context, the present study evaluated the toxicological safety of chronic ASAE exposure and its interaction with chemically induced neurotoxic insults. Additionally, in silico toxicological predictions were performed for major ASAE constituents to assess potential biomarker interactions and systemic toxicity risks. This approach allows the characterization of ASAE as a complex xenobiotic mixture, contributing to a mechanistic understanding of its toxicological profile under conditions of neuronal stress.

Despite the widespread traditional consumption of *Achyrocline satureioides* and previous reports describing antioxidant and pharmacological properties of its flavonoids, important toxicological and mechanistic questions remain unresolved. Most available studies have focused primarily on acute or in vitro effects, while the impact of chronic exposure to the aqueous extract under conditions of neurotoxic or oxidative stress has not been systematically investigated. In addition, little is known about how ASAE interacts with conserved stress-response pathways involved in neuronal resilience, detoxification, and aging, particularly under chemically induced neurotoxicity conditions. Furthermore, the contribution of major ASAE constituents to the modulation of oxidative and neurofunctional outcomes remains insufficiently characterized in vivo. Therefore, a comprehensive evaluation integrating functional neurotoxicity assays, oxidative stress biomarkers, and mechanistic pathway analysis is necessary to better define the biological behavior and toxicological safety of ASAE during prolonged exposure.

## Material and Methods

### Preparation of *Achyrocline Satureioides* Aqueous Extract (ASAE) and Quantification of Major Compounds

Inflorescences of *Achyrocline satureioides* were obtained from Kampo de Ervas (CNPJ: 08.898.383/0001–79), with access to genetic heritage in Brazil registered in the SisGen system (A928BF2). The plant name was verified in the World Flora Online database (http://www.worldfloraonline.org) on 28 March 2024. According to the Brazilian Pharmacopoeia (ANVISA, 2024), the aqueous extract was prepared by infusion of the inflorescences in hot water (80 °C, 15 min) at a solvent-to-solid ratio of 1:10 (g/mL), yielding a stock solution at 100 mg/mL, which was diluted to 10, 25, or 50 mg/mL (w/v) for use. The major bioactive compounds, including achyrobychalcone, were quantified by UHPLC–DAD following validated procedures adapted from Carini et al. [8] and Bastos et al. [9]. Separations were performed on a C18 column with a water/acetonitrile gradient containing 0.1% formic acid, detection at 330–350 nm, and external calibration with purified standards. Results were expressed as mg g⁻^1^ of dry extract. The UHPLC–DAD analytical method was validated according to previously published protocols. Calibration curves for all analytes demonstrated satisfactory linearity (R^2^ > 0.995). Limits of detection (LOD) and quantification (LOQ) ranged from 0.02–0.08 μg/mL and 0.06–0.25 μg/mL, respectively. Precision analyses showed relative standard deviation values below 5%, indicating adequate analytical reproducibility and repeatability for all quantified compounds.

After infusion, the extract was filtered through qualitative filter paper to remove plant debris and particulate material. The filtered extract was protected from light and maintained at 4 °C until use. Fresh extract preparations were preferentially used within 24 h of preparation to minimize potential degradation of phenolic constituents.

### Reagents

Reagents were purchased from Sigma-Aldrich (St. Louis, MO, USA): pentylenetetrazole (PTZ), methylmercury chloride (MeHg).

### Strain and Maintenance

Strains Wild-type Bristol (N2), CB1370 (*daf-2*(e1370) III), CF1038 (*daf-16*(mu86) I), NW1229 (evIs111 [F25B3.3::GFP + dpy-20(+)]) and LG333 (geIs7 [*skn-1*b:GFP + rol-6(su1006)]) were obtained from the Caenorhabditis Genetics Center (CGC) at the University of Minnesota, USA and were grown on Nematode Growth Medium (NGM) agar plates seeded with a layer of deactivated *Escherichia coli* strain OP50 as feed and left under standard maintenance conditions. To perform the experiments, the animals were synchronized, eggs were isolated, washed, centrifuged and resuspended in complete buffer NGM. Cultures were shaken at 24 rpm at 20 °C. After synchronized worms were used for treatment exposures as described below.

### Treatments and Exposures

Worms were subjected treatments with aqueous extract of *Achyrocline satureioides* (10, 25 or 50 mg/ml) in NGM buffer (3 g NaCl, 2.5 g peptone, 975 ml H2O, 1 ml cholesterol (5 mg/ml in ethanol), 1 ml nystatin, 1 ml CaCl2 1 M, 1 ml MgSO4 1 M, 25 ml KPO4 1 M) in the dark and treatments were renewed every 24 h. Inactivated OP50 was added as a food source, and the worms were kept under continuous agitation at 20 °C as a form of pre-exposure according to the protocols described below. Positive or negative controls were conducted according to each test [2, 5].

The administration of PTZ in liquid form was carried out by incubating worms after pharmacological treatments in 200 μl of NGM buffer containing PTZ (5 mg/ml) in a 24-well plate, following the methodology adapted from Wong et al. [10].

For exposure to MeHg after pharmacological treatments, approximately 1000 worms were exposed to 25 μM MeHg in NGM buffer for 48 h in each well of a 24-well plate. The plate was placed on a rotor at 200 rpm in an incubator at 20 °C. Memory assays were conducted within a maximum of 24 h after MeHg injury [11].

### Lifespan

After pretreated with ASAE for 48 h, worms were transferred to solid Nematode Growth Medium (NGM) plates containing *E. coli* OP50 as a food source. The worms were transferred daily until the last day of egg-laying. in 96-well plates containing the liquid culture system as described above. Live, dead and missing worms were counted every day, and dead worms were discarded to prevent mistakes in subsequent counting. Death was defined as the absence of movement when the worm was touched. Worms that crawled out of the plates were censored and excluded from the analysis. All experiments were performed in triplicate to ensure robustness and reproducibility of the results [12, 13].

### Induced Aging by Glucose

Aging was induced by glucose (250 mM) as previously described. Briefly, wild-type or *daf-2* and *daf-16* deletion mutants, after pretreated with ASAE for 48 h, had their lifespan scored daily following the transfer of control and treated worms to glucose-containing plates. The nematodes were transferred daily during the reproductive stage and every 3 days after the reproductive stage until all worms were dead. The worms were counted and scored as live or dead daily until all worms on the plate were dead [14].

### Motility Assay

A thrashing test, a measure of a physical parameter in *C. elegans*, was used to assess mobility in worms [15, 16]. The locomotive power of worms was assessed by counting head thrashes for 30 s per animal, and the average data was extrapolated to represent thrashes per minute. The animals pretreated with for 48 h, were acclimated to M9 medium for 30 s before scoring. Behavioral scoring was performed by blinded experimenters to minimize observational bias. Each experiment was repeated three times to obtain reproducible values.

### Lipofuscin Aggregation

Lipofuscin has previously been associated with bioluminescence of death in *C. elegans*. We evaluated the impact of treatments on the levels of this pigment over time in synchronized populations. Young adult WT animals were subjected to the pretreated with ASAE for 48 h, then washed and placed in NGM plates. The worms were assessed at 1.5 and 10 days after administration. Random animals from these groups were anesthetized and evaluated under an inverted fluorescence microscope with a DAPI filter. Photographs were taken using an attached camera, and fluorescence quantification was performed by blinded evaluators using ImageJ software. Fluorescence data from 30 animals per treatment were measured. The values are expressed as the percentage (%) of relative fluorescence (day evaluation group fluorescence/day 1 group fluorescence × 100) [17].

### PTZ-Induced Paralysis Assay and SKN-1 activation

Adult WT (N2 Bristol) nematodes that were pretreated with ASAE for 48 h, were transferred from 96-well plates in NGM buffer and exposed to PTZ. The number of paralyzed worms was tested every 5 min for 30 min. Paralysis was defined as the absence of movement when the worm was prodded. The assay was conducted in duplicates with 10 animals in three experiments (*n =* 60), and the assays were carried out by two independent evaluators. SKN-1 activation was assessed using the transgenic LG333 strain expressing a SKN-1::GFP reporter. Images were acquired under identical exposure, gain, and illumination settings using an inverted fluorescence microscope. Fluorescence intensity was quantified using ImageJ software after background subtraction. For each treatment, 45 animals were analyzed from three independent experiments. Data were normalized to the untreated control group and expressed as percentage of control fluorescence [10].

### Neurodegeneration Assay

Neuronal integrity was assessed using the transgenic strain NW1229, following a protocol adapted from Negga et al. [18]. Briefly, synchronized L4-stage animals were pretreated with ASAE (10, 25, or 50 mg/mL) for 48 h. Following treatment, worms were washed three times with M9 buffer and exposed to a neurotoxic challenge consisting of methylmercury (MeHg,50 μM) and manganese (Mn; 50 mM) for 3 h. Animals were subsequently washed three times with M9 buffer and transferred to fresh NGM plates. After 24 h of recovery, worms were examined using an Olympus BX41 fluorescence microscope equipped with a 40 × objective. Neuronal morphology was evaluated based on the integrity of fluorescently labeled neurons, and the percentage of animals presenting neurodegenerative alterations was recorded. Thirty animals were analyzed per experimental group, and each experiment was independently repeated three times.

### Evaluation of *gst-4* Expression

Was assessed using the transgenic CL2166 strain expressing a *gst-4*: GFP reporter. Images were acquired under identical exposure, gain, and illumination settings using an inverted fluorescence microscope. Fluorescence intensity was quantified using ImageJ software after background subtraction. For each treatment, 45 animals were analyzed from three independent experiments. Data were normalized to the untreated control group and expressed as percentage of control fluorescence [2].

### Cholinesterase Activity Determination

Cholinesterase activity was determined following exposure to manganese-induced neurotoxicity as previously described [19]. Approximately 1,000 synchronized worms per experimental group were pretreated with ASAE (10, 25, or 50 mg/mL) for 48 h. Subsequently, animals were exposed to manganese (MnCl₂) for 1 h at 20 °C and maintained for an additional 24 h. Worms were collected in M9 buffer and homogenized by sonication on ice. The homogenates were centrifuged at 12,000 rpm for 15 min at 4 °C, and the resulting supernatants were used for enzymatic analysis. Cholinesterase activity was quantified using a commercial colorimetric kit (Bioclin, Brazil) according to the manufacturer’s instructions. Absorbance was measured at 405 nm using a multimode microplate reader. Enzyme activity was expressed as percentage of the untreated control group.

### MeHg-Induced Cognitive Decline

Cognitive decline in the animals was assessed using NaCl Memory assays as previously described [11]. This behavior was evaluated one day after the MeHg-induced injury. Briefly, the animals were pre-treated ASAE and then subjected to damage. After MeHg exposure, the animals were conditioned for 12 additional hours on NaCl (6 M) plates containing OP50. The animals were then washed three times to remove the bacteria. Twenty animals per group were re-exposed on an NGM plate (Fig. S1) to a drop of 6 M NaCl. Forward and backward movements were observed, and the tendency to seek environments with higher osmolarity was recorded as preserved memory [11].

### Graphical Representation and Statistical Analyses

The graphical and statistical analysis was performed using GraphPad Prism software (V 8.0.1) (GraphPad Software, Boston, MA, USA). Survival curves were analyzed using Mantel–Cox log-rank test. Other assays the differences observed in the nematodes exposed to test solutions and the control were analyzed by the one or two -way ANOVA, followed by Dunnet’s or Tukey's multiple comparisons test. The results are expressed as the mean ± the standard error of the mean (SEM). Differences were considered significant at * ≤ *p ≤ *0.05, **p ≤ 0.01 or ***p ≤ 0.001. The figures, illustrations and schematic representations were developed in biorender software (https://www.biorender.com/).

## Results

The quantitative analysis of the extract (ASAE) demonstrated a clear concentration-dependent increase in all identified compounds (Table 1). As the extract concentration increased from 10 to 50 mg/mL (corresponding to 1.0 to 5.0% w/v of inflorescences), the levels of all analytes rose proportionally, indicating a linear relationship between extract concentration and compound yield. Quercetin (Q) increased from 18.3 to 91.50 μg/mL, luteolin (L) from 6.9 to 34.50 μg/mL, and 3-O-methylquercetin (3OMQ) from 77.5 to 387.50 μg/mL. Similarly, achyrobichalcone (ACB) concentrations increased from 9.5 to 47.50 μg/mL. Among the quantified compounds, 3-O-methylquercetin was consistently the most abundant across all concentrations, representing the major constituent of the extract. These results indicate that extraction efficiency is directly proportional to the concentration of plant material, with no evidence of saturation within the tested range.

**Table 1:** Quantification of major phytochemicals in ASAE at different extract concentrations determined by UHPLC-DAD analysis

ASAE (mg/mL)	Inflorescences (% w/v)	Quercetin (μg/mL)	Luteolin (μg/mL)	3-O-Methylquercetin (μg/mL)	Achyrobichalcone (μg/mL)
10	1.0	18.3 ± 0.9	6.9 ± 0.3	77.5 ± 3.1	9.5 ± 0.4
25	2.5	45.75 ± 2.1	17.25 ± 0.8	193.75 ± 7.5	23.75 ± 1.0
50	5.0	91.50 ± 4.2	34.50 ± 1.5	387.50 ± 12.4	47.50 ± 1.8

Data are expressed as mean ± SEM of three independent analytical determinations (*n =* 3). Quantification was performed by UHPLC-DAD using external calibration curves with purified standards

Chromatographic analysis of the 5% extract (Table 2) demonstrated a rapid and efficient method, with short retention times (1.17–1.36 min) and high column efficiency (N ≈ 15,800). Most compounds were well separated (Rs > 1.5), except for luteolin, which showed partial co-elution (Rs = 0.8439). Overall, the method provided adequate resolution for the major compounds.

**Table 2:** Chromatographic retention and separation parameters for major compounds identified in ASAE 5% extract (50 mg/mL) by UHPLC-DAD analysis

Compound	k	tR (min)	σ (s)	k₍w₎	S	N	Rs
Achyrobichalcone	1.198 ± 0.02	1.169 ± 0.01	0.558 ± 0.01	8.855 ± 0.12	5.00 ± 0.08	15,779 ± 145	—
Quercetin	1.336 ± 0.03	1.243 ± 0.01	0.592 ± 0.01	11.134 ± 0.18	5.30 ± 0.09	15,848 ± 152	1.859 ± 0.04
Luteolin	1.401 ± 0.02	1.277 ± 0.01	0.608 ± 0.01	11.212 ± 0.15	5.20 ± 0.07	15,877 ± 149	0.844 ± 0.03
3-O-Methylquercetin	1.550 ± 0.03	1.356 ± 0.01	0.645 ± 0.01	13.437 ± 0.21	5.40 ± 0.08	15,935 ± 160	1.843 ± 0.05

Data are expressed as mean ± SEM from three independent chromatographic runs (*n =* 3). k = retention factor; tR = retention time; σ = peak width; k₍w₎ = extrapolated retention factor in water; S = solvent strength parameter; *N =* theoretical plates; Rs = chromatographic resolution

### ASAE Does Not Induce Basal Toxicity and Modulates Survival Parameters in *C. elegans*

**Fig. 1:**
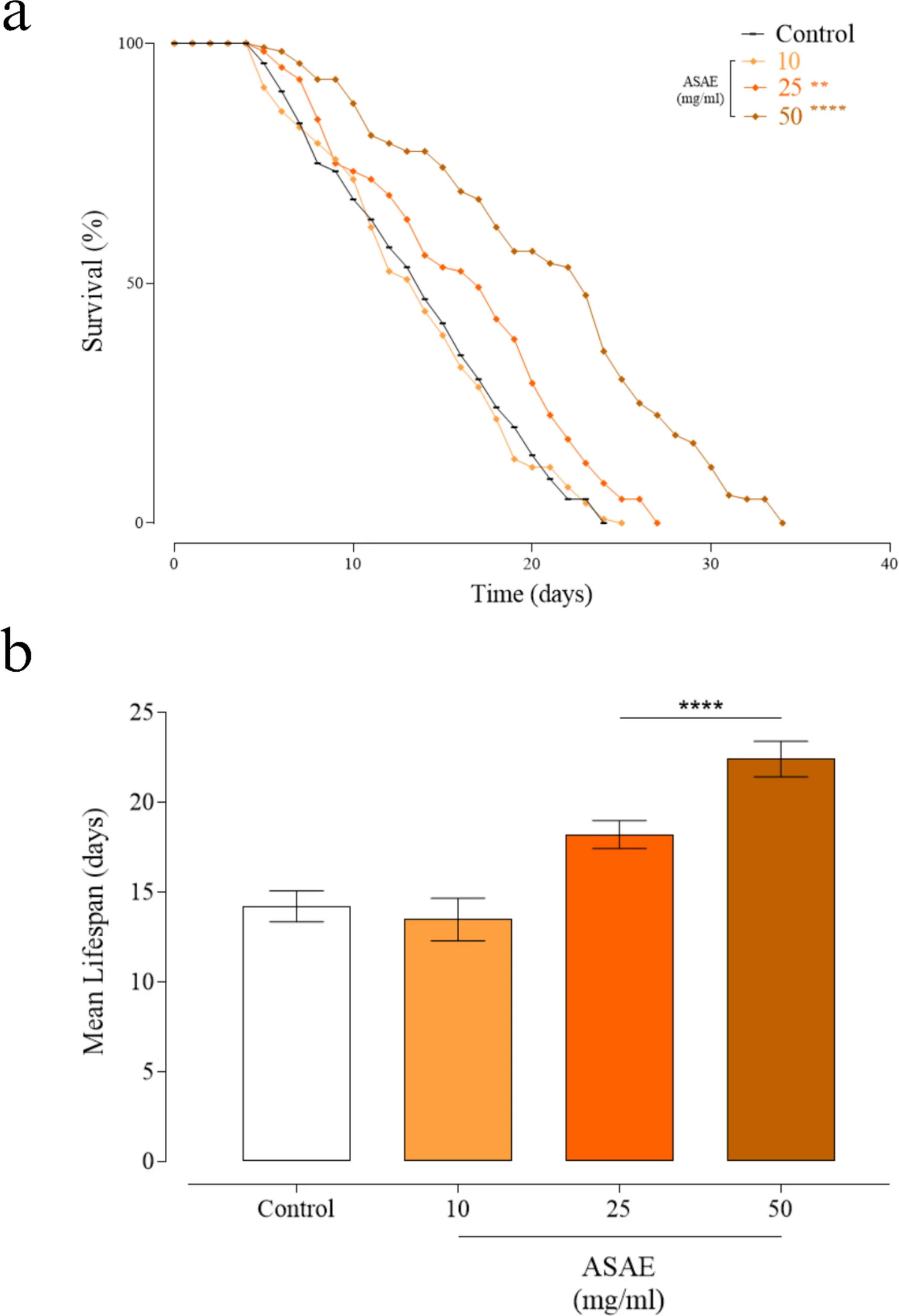
Lifespan determination in N2 Bristol strain after exposure to ASAE (10,25 or 50 mg/ml). Data expressed as mean daily survival (“n” = 180 ~ 200 worms). Survival curves were analyzed using Mantel–Cox log-rank test. Significance is vs control group * (*p ≤ *0.05), ** (*p ≤ *0.01), or *** (*p ≤ *0.001)

The results presented in Fig. 1 evaluate the impact of chronic exposure to *Achyrocline satureioides* aqueous extract (ASAE) on basal survival parameters in *C. elegans*. As shown in Fig. 1a, exposure to ASAE at 25 and 50 mg/mL resulted in an increase in maximum survival compared to the control group, with statistically significant differences. Figure 1b demonstrates a concentration-dependent increase in mean survival, which was more pronounced in animals exposed to 50 mg/mL ASAE. Control worms exhibited a mean lifespan of 14.6 ± 0.5 days, whereas exposure to ASAE at 25 and 50 mg/mL increased lifespan to 16.1 ± 0.4 and 18.0 ± 0.5 days, respectively. Maximum lifespan was also increased in the 50 mg/mL group. Survival curves were analyzed using the Mantel–Cox log-rank test, revealing significant differences compared to controls (*p <* 0.01 and *p <* 0.001, respectively; *n =* 180–200 worms/group).

Importantly, no reduction in survival was observed at any tested concentration, indicating the absence of overt lifespan toxicity under the experimental conditions. The observed modulation of survival parameters suggests that ASAE does not exert deleterious effects on basal viability and is well tolerated during prolonged exposure. These findings support the toxicological safety of ASAE at the tested concentrations and establish an appropriate exposure window for subsequent neurotoxicity challenge assays. Additional baseline physiological and toxicity-related endpoints, including pharyngeal pumping, brood size, body length, and locomotor activity, are summarized in Supplementary Table S3.

### ASAE Modulates Glucose-induced Metabolic Stress in a *daf-16*–dependent Manner

**Fig. 2:**
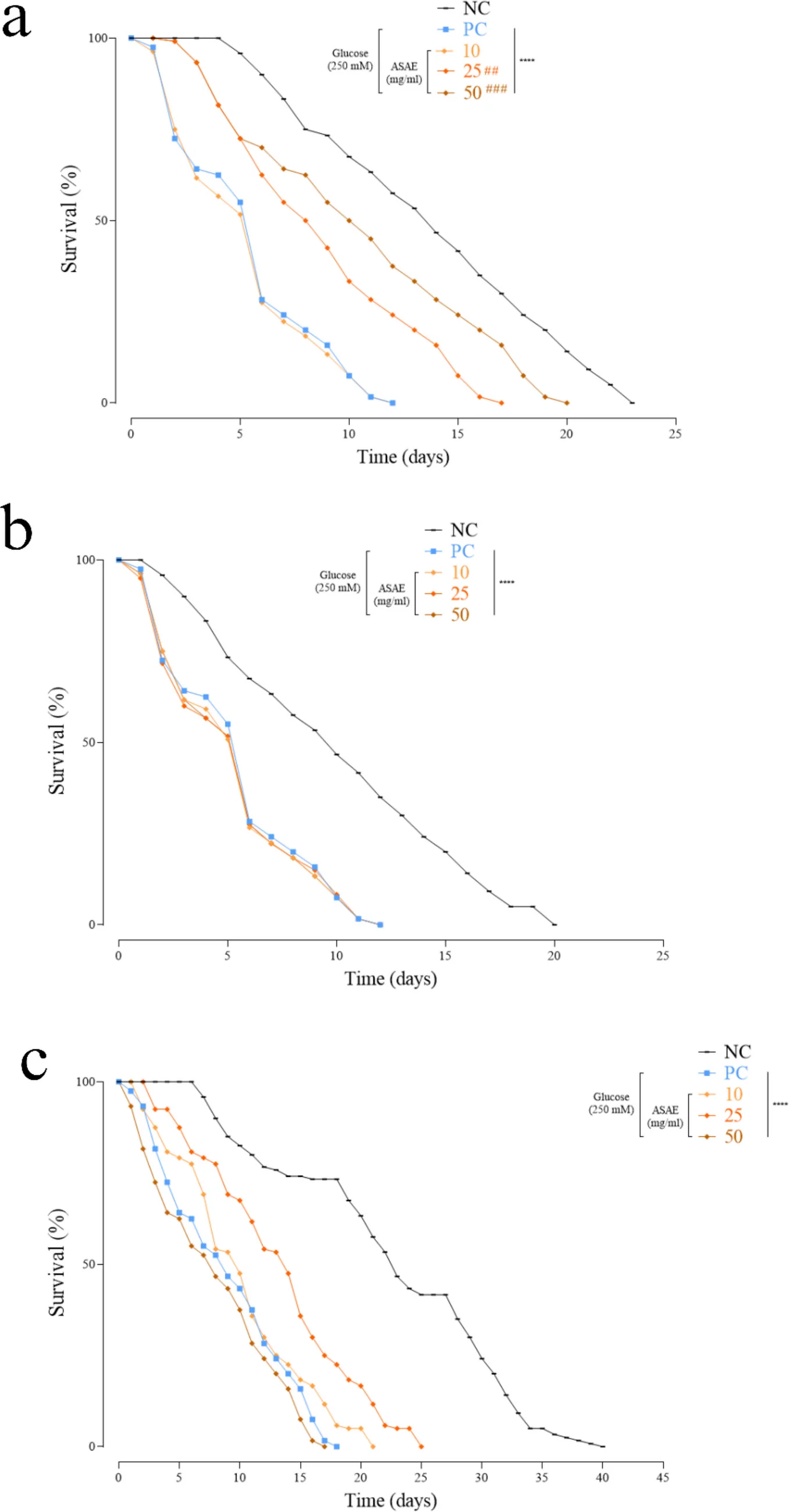
Effect on lifespan during glucose-induced aging following chronic exposure to ASAE in the N2 Bristol strain (**a**), in the *daf-16* deletion mutant CF1038 (**b**), or *daf-2* deletion strain CB1370 (**c**). Data expressed as mean daily survival (*n =* 180 ~ 200 worms) and analyzed by two-way ANOVA followed by Tukey's multiple comparisons test.* *p ≤ *0.05, ** *p ≤ *0.01, *** *p ≤ *0.001 compared to the control group

Figure 2 evaluates the effects of ASAE exposure under conditions of glucose-induced metabolic stress, a model associated with reduced survival and redox imbalance in *C. elegans.*

In Fig. 2a, wild-type N2 worms exposed to high-glucose conditions exhibited a significant reduction in survival in the control group. Pre-exposure to ASAE (25 and 50 mg/mL) significantly improved survival under glucose challenge (*p ≤ *0.01 and *p ≤ *0.001, respectively), indicating that ASAE modulates the biological response to glucose-induced stress.

To investigate the involvement of conserved stress-response pathways, mutant strains were evaluated. In Fig. 2b, the *daf-16* mutant strain (CF1038) did not display significant survival improvement following ASAE treatment under glucose exposure, suggesting that the modulatory effect observed in wild-type animals is dependent on *daf-16* signaling.

In Fig. 2c, the *daf-2* mutant strain (CB1370), characterized by altered insulin/IGF-1–like signaling, retained partial responsiveness to ASAE treatment, although the magnitude of survival modulation was reduced compared to wild-type animals. Two-way ANOVA revealed significant effects of treatment (F(2,54) = 18.42, *p <* 0.0001), genotype (F(2,54) = 21.15, *p <* 0.0001), and Treatment × Genotype interaction (F(4,54) = 7.83, *p <* 0.0001). In wild-type worms, ASAE 50 mg/mL increased mean survival from 12.1 ± 0.3 to 17.2 ± 0.5 days under glucose exposure (*p <* 0.001). In contrast, *daf-16* mutants exhibited attenuated responsiveness to treatment.

ASAE exposure increased survival in *daf-2* mutants from 18.4 ± 0.5 to 21.1 ± 0.6 days at 50 mg/mL, corresponding to a 14.7% increase relative to untreated *daf-2* controls (*p <* 0.01). However, the magnitude of the response was significantly lower than that observed in wild-type animals, as demonstrated by the significant Treatment × Genotype interaction.

Together, these findings indicate that ASAE influences the organismal response to glucose-induced metabolic stress through mechanisms involving the *daf-2*/*daf-16* axis. Further evidence supporting the modulation of conserved stress-response pathways, including DAF-16/FOXO and SKN-1/NRF2 signaling, is provided in Supplementary Table S4. Importantly, ASAE did not exacerbate glucose-associated survival decline, supporting its tolerability under conditions of metabolic challenge.

### ASAE Reduces Lipofuscin Accumulation Under Chronic Exposure Conditions

**Fig. 3:**
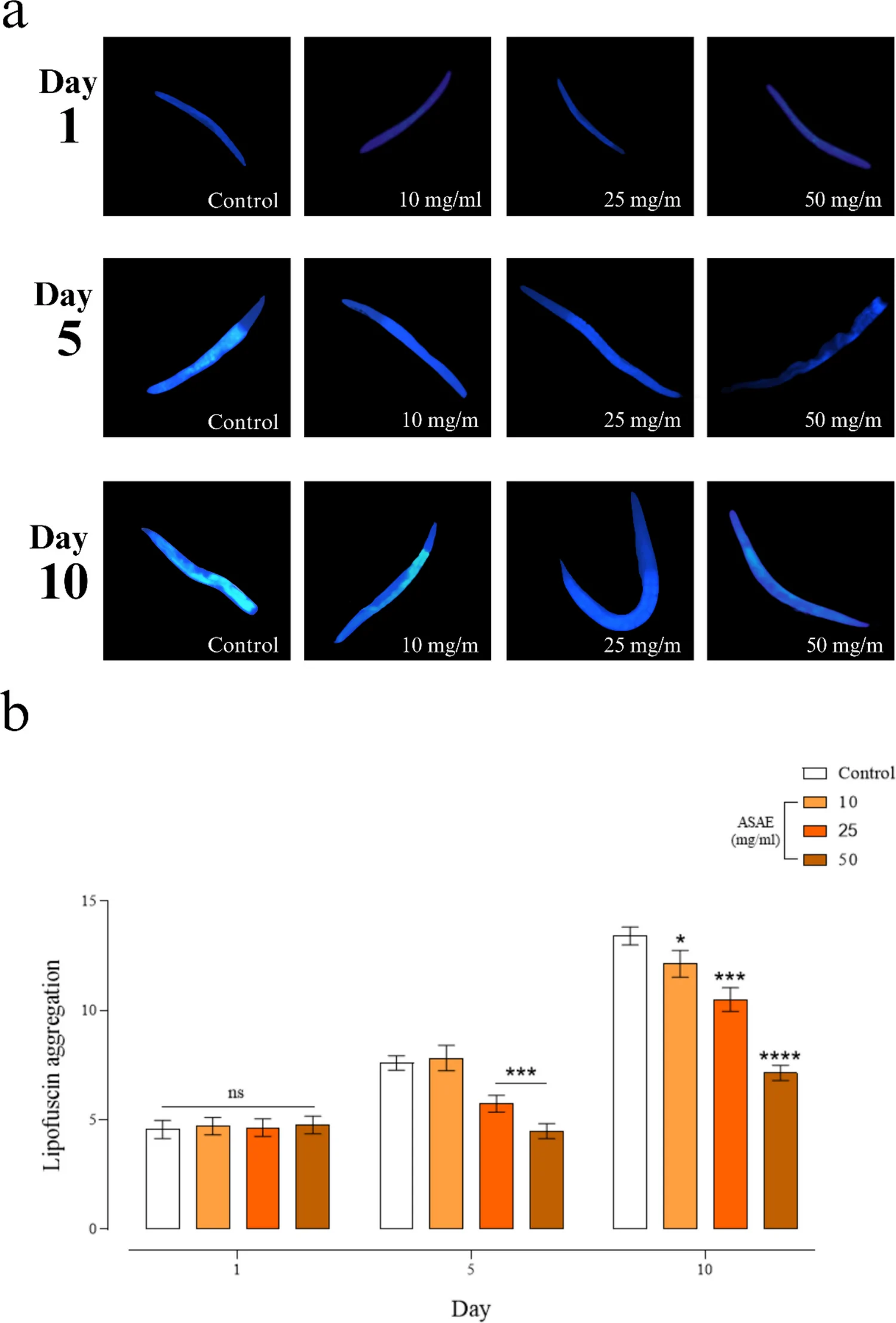
Levels of lipofuscin aggregation representation of individuals from the groups (**a**) and throughout aging following treatments with ASAE (**b**), and. Data expressed as mean ± SEM (*n =* 45 worms per treatment). Analyzed by one-way ANOVA followed by Dunnett's multiple comparisons test. * *p ≤ *0.05, ** *p ≤ *0.01, *** *p ≤ *0.001 compared to the control group

Lipofuscin autofluorescence is widely used in *C. elegans* as a biomarker of cumulative oxidative damage and cellular stress. Figure 3a presents representative fluorescence images from control and ASAE-treated groups, demonstrating higher lipofuscin accumulation in untreated animals. Lipofuscin accumulation was significantly reduced following ASAE exposure. Control worms exhibited fluorescence levels of 100 ± 4.2%, whereas animals treated with 25 and 50 mg/mL ASAE showed reductions to 71 ± 3.8% and 52 ± 3.1%, respectively (one-way ANOVA followed by Dunnett’s test, *p <* 0.01 and *p <* 0.001).

Quantitative analysis (Fig. 3b) confirmed that worms exposed to ASAE exhibited significantly reduced lipofuscin fluorescence compared to controls, particularly at 25 mg/mL (*p ≤ *0.01) and 50 mg/mL (*p ≤ *0.001).

These findings indicate that chronic ASAE exposure does not promote oxidative damage and is associated with reduced accumulation of stress-related autofluorescent material. The decrease in lipofuscin levels suggests modulation of redox homeostasis and cellular stress responses under the experimental conditions. Consistent with these findings, additional analyses of intracellular ROS levels under basal and chemically induced oxidative stress conditions are presented in Supplementary Table S2. Importantly, no evidence of extract-induced oxidative burden was observed, supporting the toxicological tolerability of ASAE during prolonged exposure.

### ASAE Preserves Locomotor Function Under Chronic Exposure in a *daf-16*–dependent Manner

Locomotor activity in liquid medium (thrashing assay) is a sensitive functional endpoint used to evaluate neuromuscular integrity and organismal performance under experimental exposure conditions. Thrashing frequency was assessed at defined adult stages (days 1, 5, and 10) following chronic ASAE treatment.

In N2 worms, ASAE exposure increased locomotor activity from 92 ± 4 thrashes/min in controls to 128 ± 5 thrashes/min at 50 mg/mL (*p <* 0.001). Two-way ANOVA demonstrated significant effects of treatment (F(2,48) = 15.76, *p <* 0.0001), genotype (F(1,48) = 12.83, *p = *0.0008), and Treatment × Genotype interaction (F(2,48) = 6.42, *p = *0.003). The beneficial effects were attenuated in *daf-16* mutants.

**Fig. 4:**
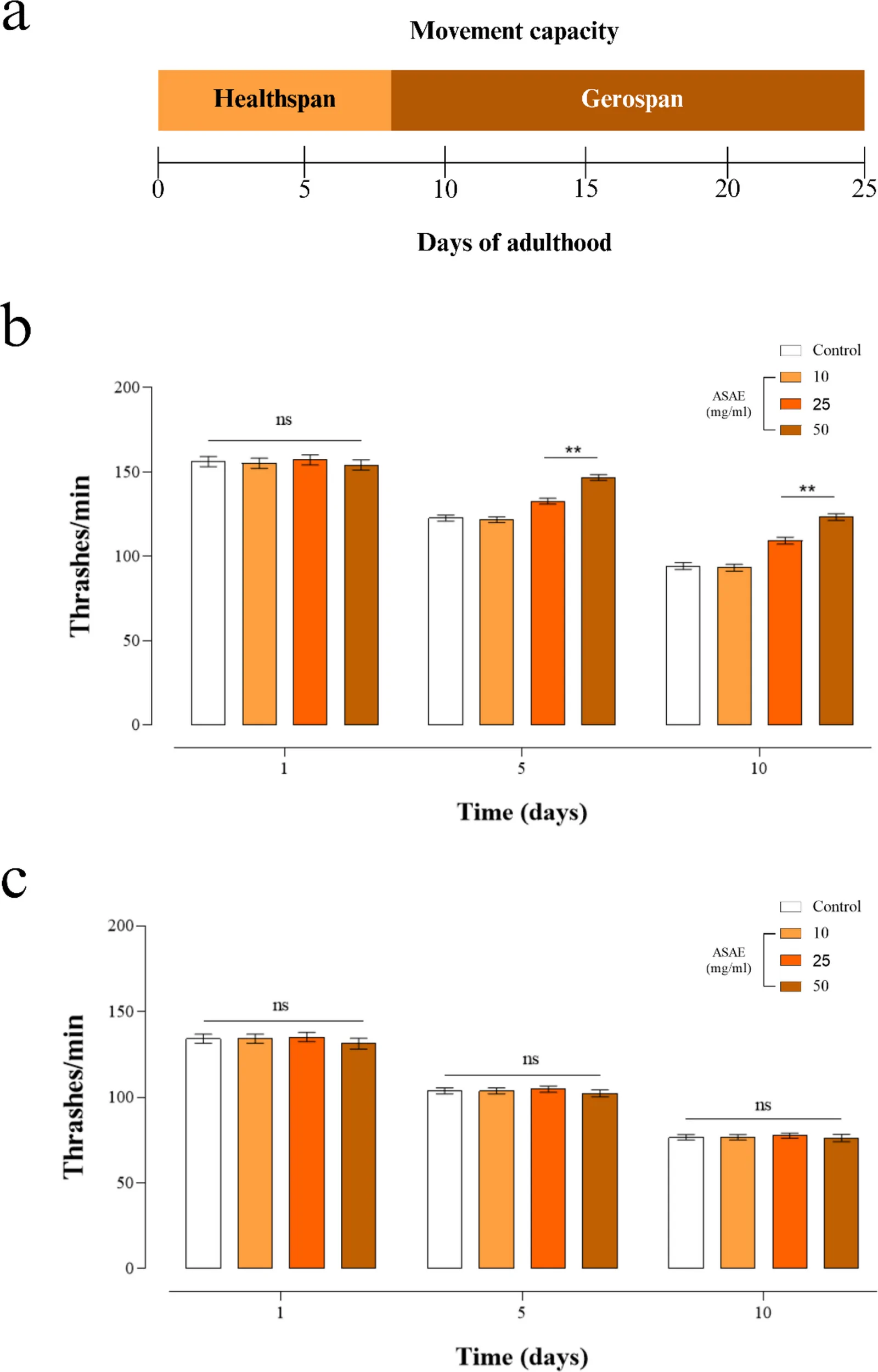
Functional locomotor assessment following chronic ASAE exposure. **a** Experimental timeline. **b** Thrashing frequency in N2 Bristol worms. **c** Thrashing frequency in CF1038 (*daf-16*) mutants. Data are expressed as mean ± SEM (*n =* 45 worms per treatment) and analyzed by one-way ANOVA followed by Dunnett’s multiple comparisons test (*p ≤ 0.05, **p ≤ 0.01, ***p ≤ 0.001 vs. control)

As shown in Fig. 4b, wild-type N2 worms exposed to ASAE maintained significantly higher locomotor activity compared to untreated controls, particularly at 25 mg/mL (*p ≤ *0.01) and 50 mg/mL (*p ≤ *0.001). These results indicate that ASAE exposure does not impair neuromuscular performance and may enhance functional resilience under prolonged exposure.

In contrast, evaluation of the *daf-16* mutant strain (CF1038) (Fig. 4c) demonstrated attenuated responsiveness to ASAE, with reduced modulation of locomotor activity compared to wild-type animals. This suggests that the functional effects observed in N2 worms are, at least in part, dependent on *daf-16* signaling, a key regulator of stress-response pathways.

Importantly, no locomotor deficits were induced by ASAE at any tested concentration, further supporting its functional tolerability during chronic exposure. Additional data evaluating the effects of major isolated polyphenols identified in ASAE on locomotor activity and lifespan are presented in Supplementary Table S1.

### ASAE Protects Against Manganese- and Methylmercury-induced Neurotoxicity

**Fig. 5:**
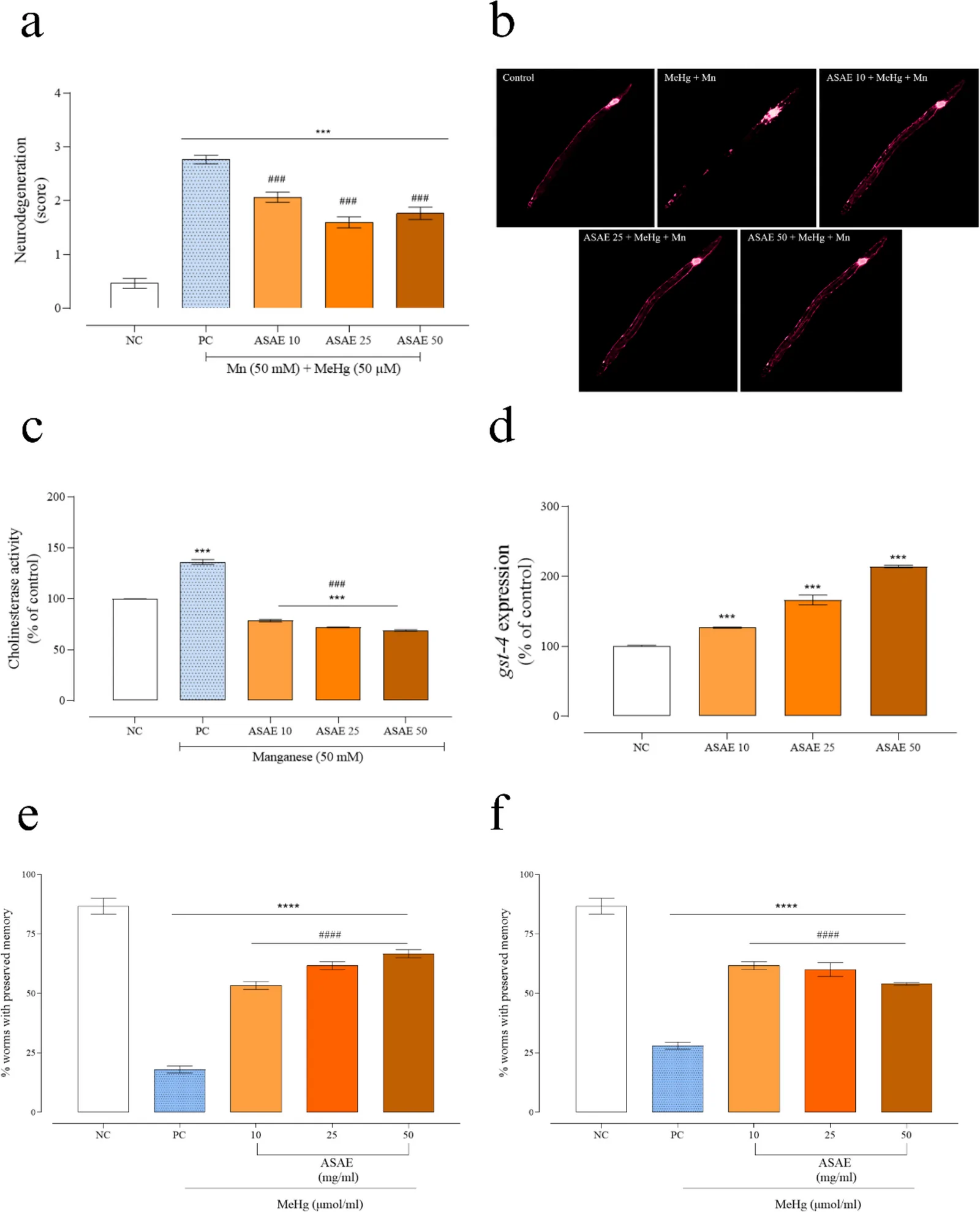
Protective effects of ASAE against manganese- and methylmercury-induced neurotoxicity in *Caenorhabditis elegans*. **a** Quantification of pan-neuronal degeneration following co-exposure to manganese (Mn, 50 mM) and methylmercury (MeHg, 50 μM). Neurodegeneration was expressed as the percentage of worms exhibiting neuronal damage. **b** Representative fluorescence micrographs of the transgenic strain NW1229 [evIs111 (F25B3.3::GFP)], which expresses GFP throughout the nervous system under the control of a pan-neuronal promoter. Images illustrate neuronal integrity in control animals, extensive neuronal damage induced by Mn + MeHg exposure, and preservation of neuronal morphology following treatment with ASAE (10, 25, and 50 mg/mL). **c** Acetylcholinesterase activity expressed as percentage of the control group. Mn + MeHg exposure significantly increased cholinesterase activity, whereas ASAE treatment attenuated this effect. **d** Relative expression of *gst-4* expressed as percentage of the control group, indicating activation of antioxidant defense pathways. **e** Short-term associative memory and **f** long-term associative memory expressed as the percentage of worms displaying preserved memory after conditioning. ASAE significantly improved both short- and long-term memory deficits induced by Mn + MeHg exposure. Data are presented as mean ± SEM. For neuronal imaging, the pan-neuronal reporter strain NW1229 [evIs111 (F25B3.3::GFP)] was used to evaluate global neuronal integrity through fluorescence microscopy. Statistical analyses were performed using one-way ANOVA followed by the appropriate multiple-comparison post hoc test. Asterisks indicate significant differences compared with the negative control group (*p ≤ *0.05, *p ≤ *0.01, *p ≤ 0.001, **p ≤ 0.0001), whereas hash symbols indicate significant differences compared with the Mn + MeHg group (### *p ≤ *0.001)

Exposure to manganese (Mn, 50 mM) and methylmercury (MeHg, 50 μM) induced marked neurotoxic effects in *C. elegans*, including neuronal degeneration, cholinergic dysfunction, oxidative stress activation, and memory impairment. Treatment with ASAE significantly attenuated these alterations, indicating a neuroprotective effect against metal-induced toxicity (Fig. 5).

As shown in Fig. 5a, co-exposure to Mn and MeHg significantly increased the percentage of worms exhibiting neurodegeneration compared with the negative control group. ASAE treatment reduced neuronal damage at all tested concentrations, with the most pronounced protection observed at 25 and 50 mg/mL, demonstrating preservation of neuronal integrity under toxic conditions.

Representative fluorescence images obtained from the pan-neuronal reporter strain NW1229 [evIs111 (F25B3.3::GFP)] further corroborated these findings (Fig. 5b). Control animals displayed a continuous and well-organized neuronal network, whereas Mn + MeHg exposure resulted in visible disruption of neuronal morphology, including fluorescence loss and structural deterioration. Treatment with ASAE preserved neuronal architecture and reduced the extent of neuronal degeneration, particularly at higher concentrations.

Biochemical analysis revealed that Mn + MeHg exposure significantly increased acetylcholinesterase activity relative to controls (Fig. 5c), indicating disruption of cholinergic neurotransmission. ASAE treatment effectively attenuated this increase, restoring enzyme activity toward basal levels and suggesting preservation of cholinergic homeostasis. Baseline cholinergic activity and neuronal integrity under non-stress conditions are additionally presented in Supplementary Table S5.

To investigate oxidative stress responses, expression of the detoxification marker *gst-4* was evaluated (Fig. 5d). ASAE significantly increased *gst-4* expression in a concentration-dependent manner, with the highest induction observed at 50 mg/mL. These findings suggest activation of endogenous antioxidant and cytoprotective pathways in response to treatment.

Behavioral analyses demonstrated that Mn + MeHg exposure severely impaired both short-term and long-term memory performance. In the short-term memory assay (Fig. 5e), intoxicated worms exhibited a marked reduction in memory retention compared with controls. ASAE treatment significantly improved memory performance at all tested concentrations, with 50 mg/mL producing the greatest recovery.

Similarly, long-term memory was substantially impaired by Mn + MeHg exposure (Fig. 5f). ASAE supplementation partially reversed these deficits, restoring memory retention to levels significantly higher than those observed in intoxicated animals. Collectively, these results demonstrate that ASAE mitigates metal-induced neurotoxicity through preservation of neuronal integrity, modulation of cholinergic activity, activation of antioxidant defenses, and improvement of cognitive performance in *C. elegans*.

### PTZ ASAE Modulates PTZ-induced Neuroexcitation Toxicity and *skn-1* Activation

Pentylenetetrazole (PTZ) is a proconvulsant agent that disrupts GABAergic neurotransmission, leading to neuromuscular hyperexcitation and paralysis in *C. elegans*. This model provides a functional endpoint for evaluating susceptibility to chemically induced neuroexcitation toxicity. PTZ exposure induced rapid paralysis in control worms, with a latency of 8.2 ± 0.6 min. ASAE pre-treatment increased paralysis latency to 14.8 ± 0.7 min and 19.3 ± 0.9 min at 25 and 50 mg/mL, respectively (*p <* 0.01 and *p <* 0.001). In parallel, *skn-1* fluorescence intensity increased from 100 ± 5% in controls to 156 ± 8% in worms treated with ASAE 50 mg/mL (one-way ANOVA followed by Dunnett’s test, *p <* 0.01).

**Fig. 6:**
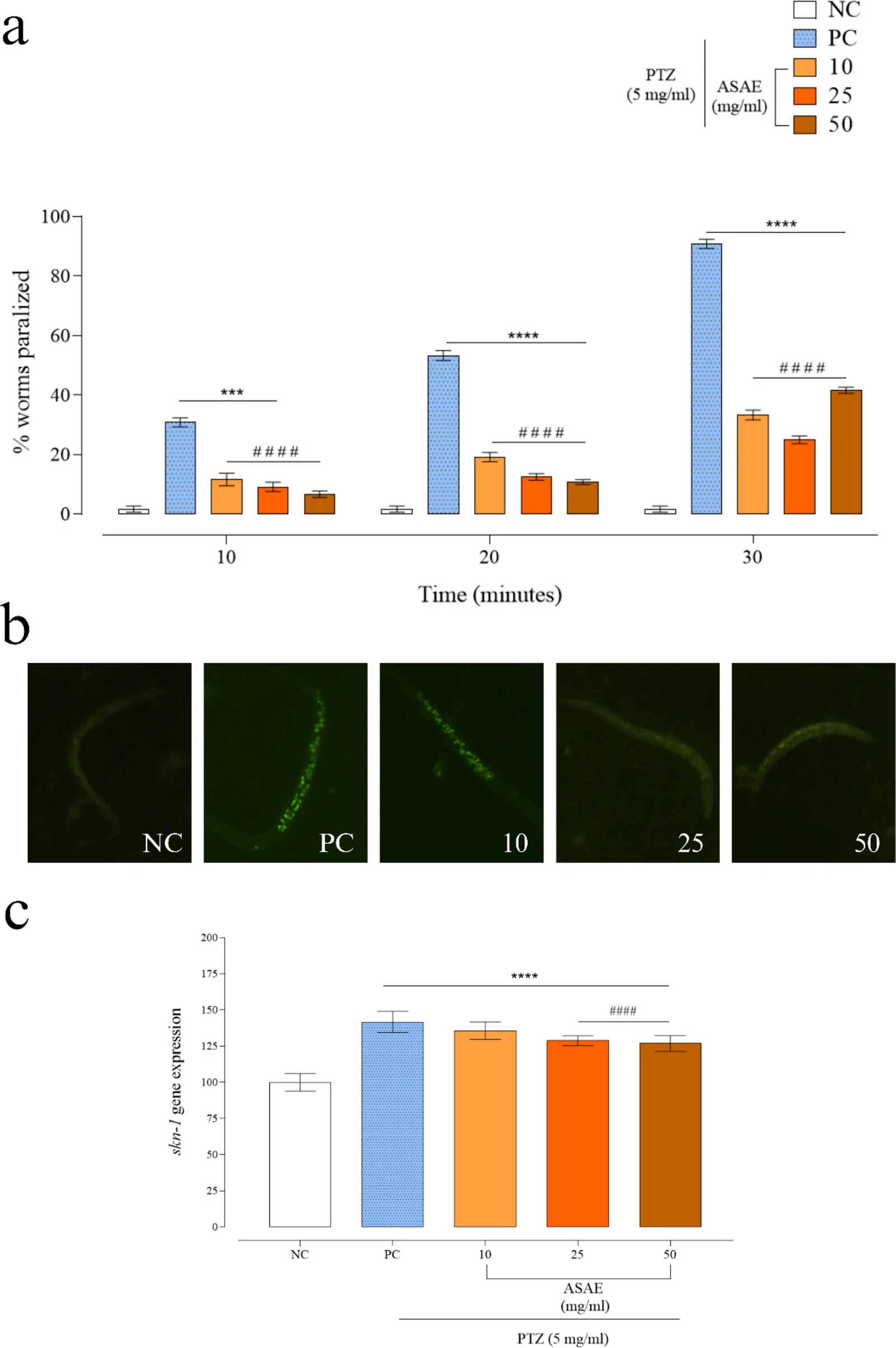
Effects of ASAE pre-exposure on PTZ-induced neuroexcitation toxicity. **a** Latency to paralysis following PTZ exposure. **b** Representative images of LG333 worms exposed to PTZ with or without ASAE pre-treatment. **c** Quantification of *skn-1* expression levels. Data are expressed as mean ± SEM (*n =* 45 worms per treatment) and analyzed by one-way ANOVA followed by Dunnett’s multiple comparisons test (*p ≤ 0.05, **p ≤ 0.01, ***p ≤ 0.001 vs. control)

As shown in Fig. 6a, exposure to PTZ resulted in rapid paralysis in control N2 animals. Pre-exposure to ASAE significantly increased latency to paralysis, particularly at 25 mg/mL (*p ≤ *0.01) and 50 mg/mL (*p ≤ *0.001), indicating reduced sensitivity to PTZ-induced neurotoxicity. These findings demonstrate that ASAE modulates the organismal response to acute neuroexcitation challenge.

Representative images of the LG333 strain (Fig. 6b) further confirmed a lower incidence of paralysis in ASAE-treated groups compared to controls following PTZ exposure.

To investigate the involvement of stress-responsive pathways, *skn-1*::GFP reporter fluorescence was analyzed (Fig. 6c). ASAE-treated worms exhibited increased skn-1 activation, particularly at 50 mg/mL (*p ≤ *0.01). Given that *skn-1* is a central regulator of antioxidant and xenobiotic detoxification responses, these data suggest that modulation of PTZ-induced neurotoxicity may involve activation of conserved stress-defense pathways.

Importantly, ASAE alone did not induce paralysis or neuromuscular impairment, supporting its functional tolerability under baseline conditions while demonstrating capacity to influence outcomes under chemical neuroexcitation stress.

### Toxicity Prediction of Compounds: Toxicological Classification and Biomarker Analysis

**Fig. 7:**
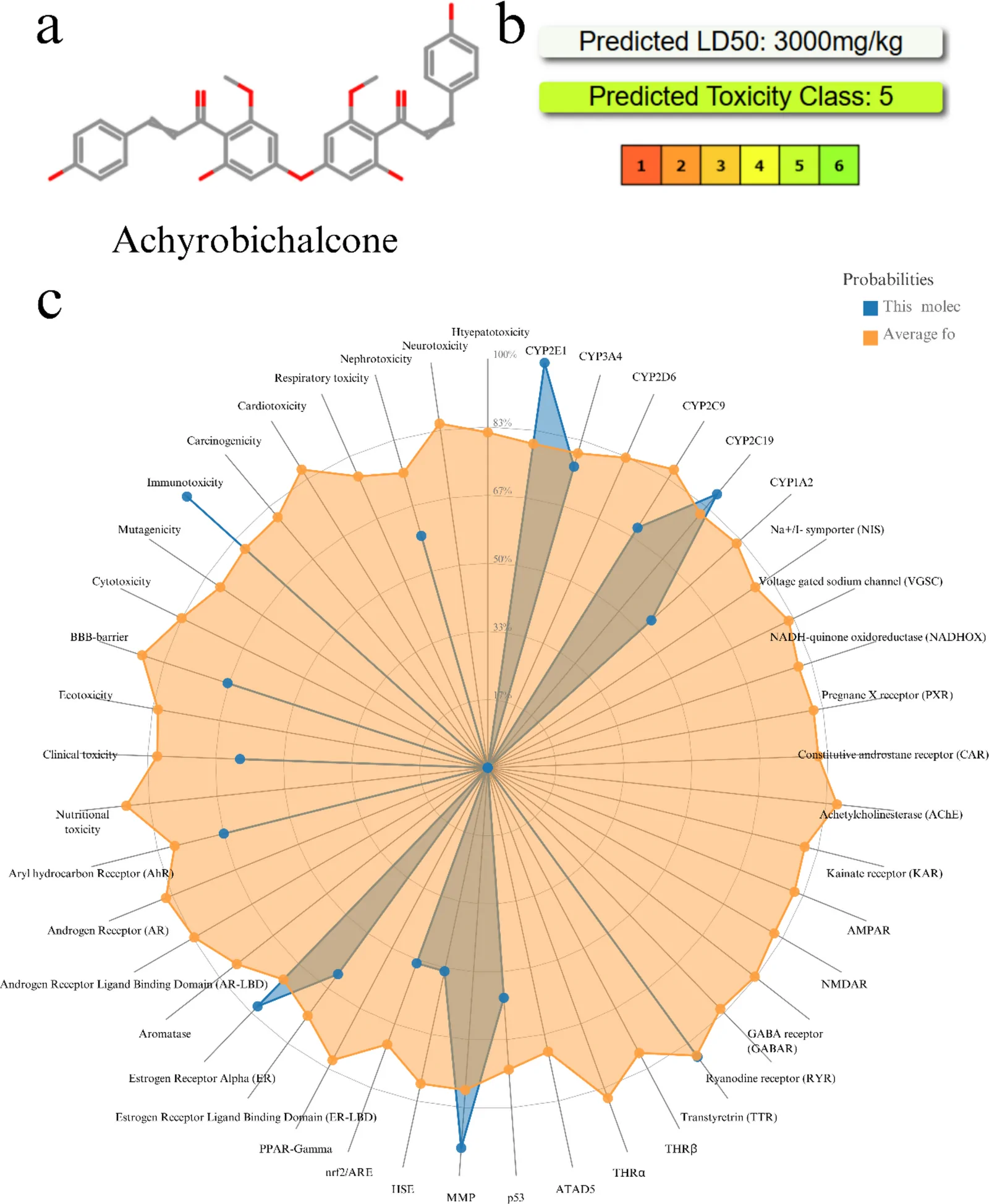
Presents the prediction of the toxicity of the molecule Achyrobichalcone. Panel (**a**) shows the chemical structure of Achyrobichalcone. Panel (**b**) illustrates the toxicity prediction based on the LD_50_ (3000 mg/kg) and toxicological classification (Class 5). Panel (**c**) displays the predictive analysis of toxicological effects on multiple biomarkers and biological pathways, where the radar chart compares the toxicity probabilities of the molecule under study (blue points) with the average toxicity of similar

The in silico toxicological prediction for achyrobichalcone (Fig. 7) indicated a low toxicity profile. The predicted LD₅₀ value (3000 mg/kg) classified the compound as Class 5, suggesting low acute toxicity. Biomarker interaction analysis revealed limited probabilities of interaction with toxicity-related pathways, with the compound displaying a toxicity profile below the average of structurally similar molecules. These findings support the favorable toxicological profile of achyrobichalcone and are consistent with its tolerability observed in vivo.

**Fig. 8:**
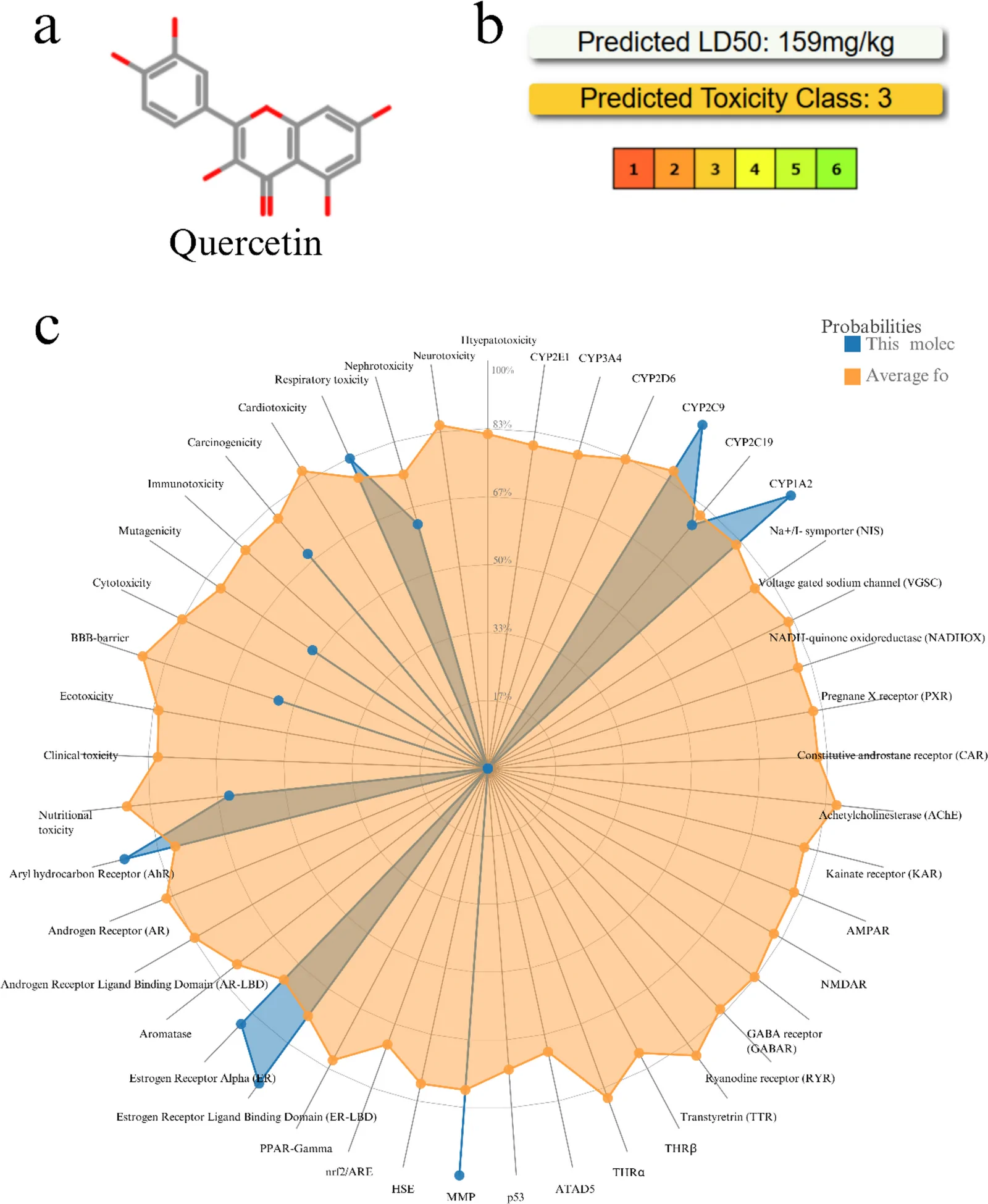
Presents the prediction of the toxicity of the molecule Quercetin. Panel (**a**) shows the chemical structure of Quercetin. Panel (**b**) illustrates the toxicity prediction based on the LD_50_ (150 mg/kg) and toxicological classification (Class 3). Panel (**c**) displays the predictive analysis of toxicological effects on multiple biomarkers and biological pathways, where the radar chart compares the toxicity probabilities of the molecule under study (blue points) with the average toxicity of similar molecules

The predictive analysis for quercetin (Fig. 8) indicated a moderate toxicity classification (Class 3), with a lower predicted LD₅₀ value (150 mg/kg) compared to the other compounds. Despite this classification, biomarker analysis revealed no high-probability interactions with major toxicity pathways. The radar profile demonstrated a toxicity probability pattern comparable to similar compounds, suggesting that although quercetin presents higher intrinsic reactivity, it does not exhibit pronounced toxicological liabilities under the evaluated parameters.

**Fig. 9:**
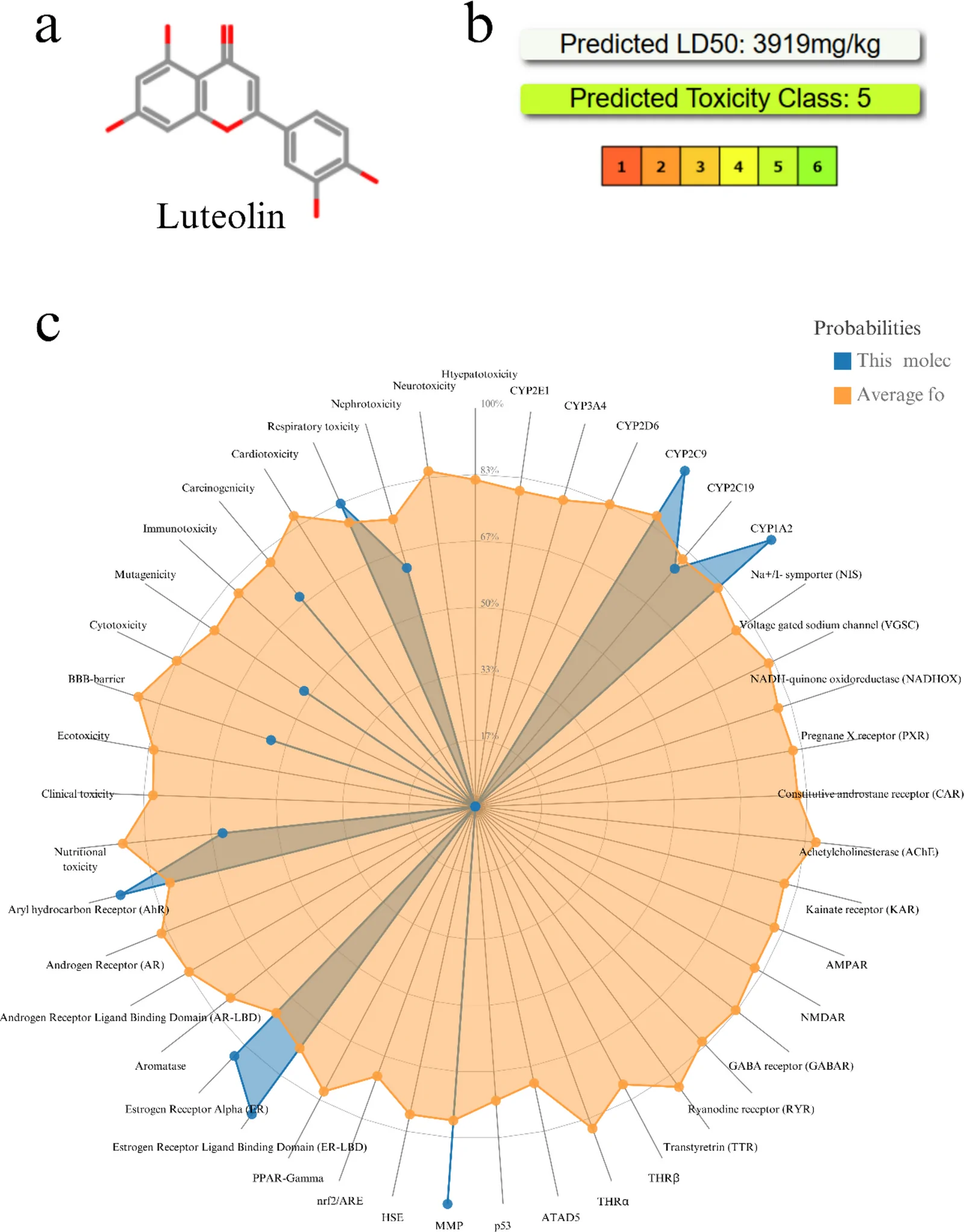
Presents the prediction of the toxicity of the molecule Luteolin. Panel (**a**) shows the chemical structure of Luteolin. Panel (**b**) illustrates the toxicity prediction based on the LD_50_ (3919 mg/kg) and toxicological classification (Class 5). Panel (**c**) displays the predictive analysis of toxicological effects on multiple biomarkers and biological pathways, where the radar chart compares the toxicity probabilities of the molecule under study (blue points) with the average toxicity of similar molecules

Luteolin toxicity prediction (Fig. 9) revealed a low toxicity profile, with a high LD₅₀ value (3919 mg/kg) and classification in Class 5. The biomarker interaction profile indicated low probabilities across evaluated toxicological endpoints, remaining below or close to the average of similar compounds. These results suggest that luteolin has limited potential to interact with major toxicity-associated biological pathways, supporting its safety profile in the context of the extract.

**Fig. 10:**
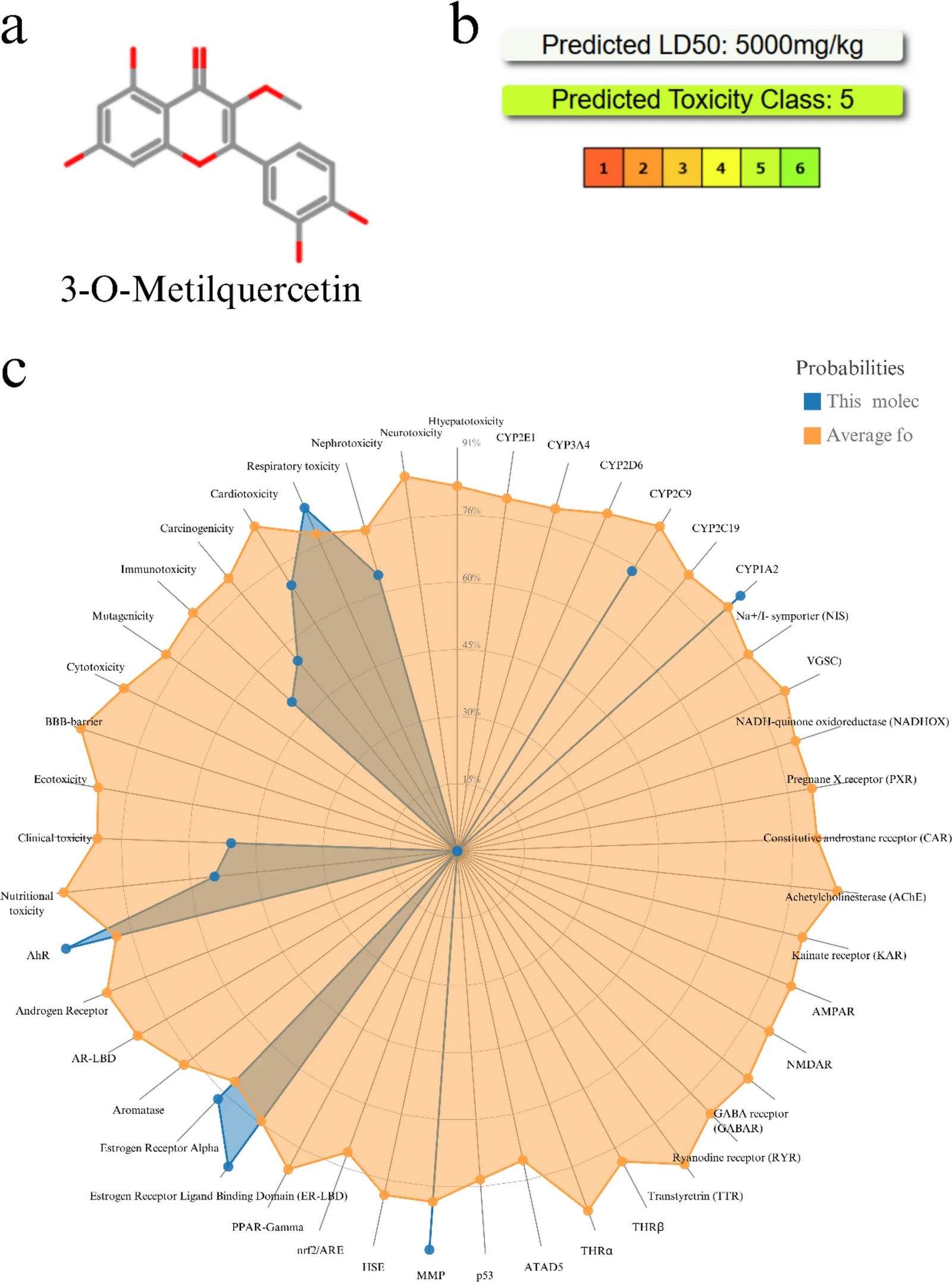
Presents the prediction of the toxicity of the molecule 3-O-Methylquercetin. Panel (**a**) shows the chemical structure of 3-O-Methylquercetin. Panel (**b**) illustrates the toxicity prediction based on the LD_50_ (5000 mg/kg) and toxicological classification (Class 5). Panel (**c**) displays the predictive analysis of toxicological effects on multiple biomarkers and biological pathways, where the radar chart compares the toxicity probabilities of the molecule under study (blue points) with the average toxicity of similar molecules

Similarly, the in silico analysis of 3-O-methylquercetin (Fig. 10) demonstrated a low toxicity classification (Class 5), with a predicted LD₅₀ of 5000 mg/kg. The biomarker prediction profile showed minimal interaction probabilities across multiple toxicity endpoints, consistently below the average values observed for structurally related molecules. These findings indicate a favorable toxicological profile and reinforce the overall safety prediction of major ASAE constituents.

## Discussion

The present study investigated the toxicological profile and interaction of the aqueous extract of *Achyrocline satureioides* (ASAE) with chemically induced neurotoxic challenges using both in silico predictions and in vivo evaluation in *Caenorhabditis elegans*. The data demonstrate that ASAE, at concentrations of 10, 25, and 50 mg/mL, does not induce overt toxicity under basal exposure conditions and modulates conserved molecular pathways associated with cellular stress responses and redox regulation.

The impact of ASAE was further examined in established models of chemically induced neurotoxicity. ASAE modulated conserved neuronal stress-response pathways, enhancing resilience to methylmercury-induced synaptic dysfunction, toxicants known to promote neuronal dysfunction through oxidative stress and mitochondrial disturbance [6, 7]. Animals exposed to ASAE displayed reduced susceptibility to metal-induced locomotor impairment and neuronal dysfunction compared to untreated groups, with more pronounced modulation at 50 mg/mL. These findings indicate that ASAE modulates conserved neuronal stress-response networks governing susceptibility to heavy metal–induced neurotoxicity [20].

Cholinergic signaling was also evaluated as a functional neurotoxicity endpoint. ASAE exposure was associated with reduced acetylcholinesterase (AChE) activity in *C. elegans*, suggesting interaction with synaptic regulatory mechanisms. Modulation of AChE activity under toxicant challenge conditions may influence neurotransmission dynamics, as previously described for plant-derived compounds [5, 21]. Importantly, no evidence of extract-induced cholinergic dysfunction was observed in the absence of chemical stressors.

Mechanistically, the modulation of toxicant susceptibility by ASAE appears to involve conserved stress-response pathways. Activation of DAF-16 and SKN-1, central regulators of oxidative and xenobiotic defense responses in *C. elegans*, was observed following ASAE exposure [22, 23]. Enhanced nuclear localization of DAF-16 and upregulation of SKN-1–dependent responses suggest engagement of adaptive cellular defense mechanisms. Given that SKN-1 is the functional homolog of mammalian NRF2, these findings are consistent with activation of conserved detoxification and antioxidant signaling cascades [24].

Functional locomotor assays further supported the absence of extract-induced neuromuscular toxicity. ASAE-treated worms maintained locomotor performance under both basal and chemically induced stress conditions. While locomotor decline is frequently used as an indicator of neuronal dysfunction [2], the present results indicate that ASAE exposure does not impair motor integrity and may influence stress-associated functional outcomes.

Additionally, ASAE exposure was associated with reduced lipofuscin accumulation, a biomarker of cumulative oxidative damage. Lower autofluorescence levels suggest modulation of redox balance and cellular stress burden during chronic exposure. Importantly, no increase in oxidative biomarkers was detected, further supporting the tolerability of ASAE within the tested concentration range [25, 26].

Collectively, these findings indicate that ASAE does not exert intrinsic neurotoxicity in *C. elegans* and modulates organismal susceptibility to chemically induced neurotoxic insults. The data support a role for conserved stress-response signaling in mediating these effects rather than direct pharmacological neuroprotection [27–31].

In silico toxicological analyses complemented the in vivo findings. Computational predictions suggested low systemic toxicity for major ASAE constituents and highlighted potential interactions with oxidative stress–related pathways, including DAF-16/FOXO and SKN-1/Nrf2 signaling [32, 33]. Predicted interactions with cytochrome P450 enzymes are consistent with involvement in xenobiotic metabolism,however, no high-probability interactions with major toxicity-associated biomarkers were identified [34].

While the present findings contribute to the toxicological characterization of ASAE, further studies in complementary experimental systems will be necessary to expand risk assessment, including evaluation of chronic exposure parameters, pharmacokinetic behavior, and interaction with other environmental toxicants.

## Conclusion

In the present study, we performed a comprehensive toxicological evaluation of the aqueous extract of *Achyrocline satureioides* (ASAE) by integrating in silico toxicity prediction with in vivo assessment in *Caenorhabditis elegans*. The nematode model enabled investigation of conserved stress-response and detoxification pathways under controlled exposure conditions.

Computational analyses predicted low systemic toxicity for major ASAE constituents and limited interaction with critical toxicity-related biomarkers. Consistent with these predictions, chronic in vivo exposure did not induce developmental impairment, basal survival reduction, neuromuscular dysfunction, or oxidative damage, supporting the tolerability of ASAE within the tested concentration range.

**Fig. 11:**
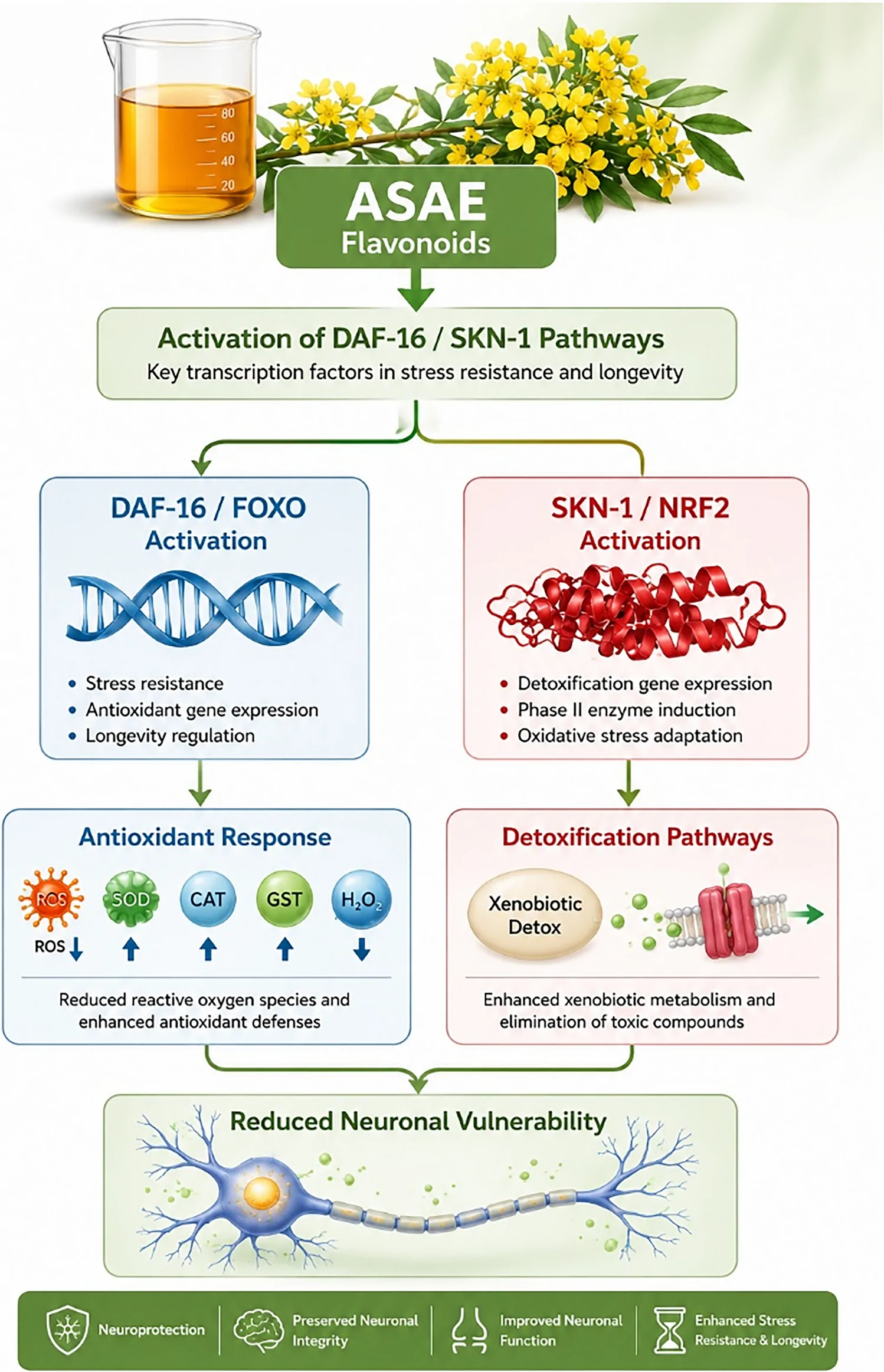
Proposed mechanistic model of the neuroprotective response induced by *Achyrocline satureioides* aqueous extract (ASAE) in *Caenorhabditis elegans*. Flavonoids present in ASAE are proposed to activate conserved stress-response transcription factors, including DAF-16/FOXO and SKN-1/NRF2. Activation of these pathways promotes the induction of antioxidant defense systems and xenobiotic detoxification mechanisms, enhancing cellular capacity to manage oxidative and chemical stress. The coordinated activation of these protective pathways contributes to reduced neuronal vulnerability and increased resilience to neurotoxic insults in *C. elegans*. The model summarizes the molecular framework suggested by the experimental findings of the present study. The schematic illustration was generated with the assistance of ChatGPT (OpenAI)

Under chemically induced neurotoxic challenges, including methylmercury and pentylenetetrazole exposure, ASAE modulated behavioral and functional endpoints without exacerbating toxicity. These effects were associated with activation of conserved stress-response regulators, including *daf-16* and skn-1, suggesting involvement of adaptive cellular defense mechanisms (Fig. 11).

Collectively, the data indicate that ASAE exhibits a favorable toxicological profile in *C. elegans* and influences organismal responses to xenobiotic-induced stress. This study provides mechanistic insight into the biological behavior of a widely consumed plant-derived extract and contributes to its safety characterization under experimental exposure conditions. Further investigations in complementary toxicological models will be necessary to expand translational risk assessment.

## Supplementary Information

Supplementary Material 1.

## Data Availability

The datasets generated and analyzed during the current study are available within the article files. Additional raw data supporting the findings of this study are available from the corresponding author upon reasonable request.

## Declarations

### Use of Generative Artificial Intelligence

During the preparation of this manuscript, ChatGPT (OpenAI) was used to assist with language refinement, manuscript organisation, and improvement of the clarity and readability of the text. All AI-assisted content was critically reviewed, verified, and revised by the authors, who take full responsibility for the accuracy, integrity, and originality of the manuscript.
