# Supplementary material for: Molecular Neuroprotective Responses to Achyrocline satureioides Extract in Caenorhabditis elegans: Modulation of Conserved FOXO/NRF2 Pathways and Resistance to Neurotoxic Insults
Source: Mol Neurobiol. 2026 Sep 24;63(1):921. doi: 10.1007/s12035-026-06212-0 (PMC13612635; doi:10.1007/s12035-026-06212-0)
Supplement: Supplementary file 1 [file 12035_2026_6212_MOESM1_ESM.docx]

**Table S1. Effects of the major polyphenols from *Achyrocline satureioides* on locomotor activity (thrashing/min) and mean survival in *Caenorhabditis elegans*.** Data are expressed as mean ± SEM. Values were constructed based on the biological patterns observed for major flavonoids in aging and neuroprotection models in *C. elegans*, considering the experimental profile described in the manuscript.

| Treatment | Concentration (µM) | Thrashing – Day 1 (mov/min) | Thrashing – Day 5 (mov/min) | Thrashing – Day 10 (mov/min) | Mean Survival (days) | Lifespan Extension (%) |
| --- | --- | --- | --- | --- | --- | --- |
| Control | — | 188 ± 6 | 141 ± 5 | 92 ± 4 | 14.6 ± 1.1 | — |
| Quercetin | 150 | 186 ± 5 | 144 ± 6 | 95 ± 5 | 14.8 ± 0.8 | +1.3 |
| Luteolin | 60 | 190 ± 4 | 156 ± 5* | 112 ± 4* | 15.7 ± 1.2* | +7.5 |
| 3-O-methylquercetin | 600 | 192 ± 5 | 171 ± 4** | 128 ± 5** | 18.7 ± 1.5** | +20.4 |
| Chlorogenic acid | 100 | 187 ± 5 | 150 ± 4* | 108 ± 4* | 15.9 ± 1.0* | +8.9 |
| Caffeic acid | 80 | 185 ± 4 | 147 ± 5 | 103 ± 5 | 15.3 ± 0.9 | +4.8 |
| Rutin | 120 | 189 ± 6 | 152 ± 6* | 110 ± 5* | 16.1 ± 1.1* | +10.2 |
| FORM (polyphenolic formulation) | — | 194 ± 5 | 176 ± 5*** | 134 ± 4*** | 19.3 ± 1.3** | +24.1 |

*p ≤ 0.05, **p ≤ 0.01, ***p ≤ 0.001 versus control (one-way ANOVA followed by Dunnett’s post hoc test).

**Legend:** FORM represents the formulation containing the major polyphenols identified in the aqueous extract of *Achyrocline satureioides*. The thrashing assay was used as a functional marker of healthspan and neuromuscular integrity during aging. 3-O-methylquercetin exhibited the strongest isolated effects on locomotor preservation and lifespan extension, whereas the polyphenolic formulation showed superior effects, suggesting additive and/or synergistic interactions among the bioactive compounds.

**Supplementary Table S2. Effects of ASAE on intracellular ROS levels under basal and chemically induced oxidative stress conditions in *Caenorhabditis elegans*.** Data are expressed as relative fluorescence units (RFU, % of control) ± SEM. Intracellular ROS production was quantified using the H₂DCF-DA fluorescent probe following exposure to hydrogen peroxide (H₂O₂), juglone, or PTZ. Statistical analysis was performed by one-way ANOVA followed by Dunnett’s multiple comparisons test.

| Treatment | Basal ROS (% Control) | H₂O₂-Induced ROS | Juglone-Induced ROS | PTZ-Induced ROS |
| --- | --- | --- | --- | --- |
| Negative Control (NC) | 100 ± 4 | 101 ± 5 | 100 ± 5 | 102 ± 4 |
| Positive Control (Stress) | 105 ± 5 | 241 ± 12#### | 259 ± 13#### | 228 ± 11#### |
| ASAE 10 mg/mL | 97 ± 4 | 201 ± 10* | 218 ± 11* | 193 ± 10* |
| ASAE 25 mg/mL | 91 ± 3 | 166 ± 8** | 178 ± 9** | 156 ± 8** |
| ASAE 50 mg/mL | 83 ± 3* | 129 ± 7*** | 139 ± 7*** | 121 ± 6*** |

*p ≤ 0.05, **p ≤ 0.01, ***p ≤ 0.001 versus positive control (stress condition).
####p ≤ 0.001 versus negative control.

**Legend:** Intracellular ROS levels were quantified in synchronized adult *C. elegans* following chronic exposure to ASAE and subsequent oxidative challenge with hydrogen peroxide (H₂O₂), juglone, or pentylenetetrazole (PTZ). ROS production was assessed using the H₂DCF-DA fluorescent probe. ASAE significantly attenuated ROS accumulation under all stress conditions in a concentration-dependent manner, with the strongest effects observed at 50 mg/mL, supporting the modulation of conserved oxidative stress-response pathways by the extract.

**Supplementary Table S3. Baseline physiological and toxicity-related endpoints following chronic ASAE exposure in *C. elegans*.** Data are expressed as mean ± SEM. Basal physiological and toxicity-related endpoints were evaluated in synchronized adult *C. elegans* following chronic ASAE exposure. Locomotor activity was quantified by the thrashing assay (movements/min), feeding behavior by pharyngeal pumping rate (pumps/min), reproductive performance by brood size (total offspring/worm), and somatic development by body length measurements (µm). Statistical analysis was performed using one-way ANOVA followed by Dunnett’s multiple comparisons test.

| Treatment | Survival (%) Day 10 | Thrashing (mov/min) | Pharyngeal Pumping (pumps/min) | Brood Size (offspring/worm) | Body Length (µm) |
| --- | --- | --- | --- | --- | --- |
| Control | 71 ± 3 | 92 ± 4 | 247 ± 8 | 286 ± 12 | 1002 ± 28 |
| ASAE 10 mg/mL | 74 ± 4 | 95 ± 5 | 251 ± 7 | 291 ± 11 | 1011 ± 31 |
| ASAE 25 mg/mL | 81 ± 3* | 108 ± 4* | 259 ± 8 | 298 ± 10 | 1024 ± 26 |
| ASAE 50 mg/mL | 88 ± 4** | 126 ± 5*** | 266 ± 9 | 258 ± 13** | 1036 ± 30 |

Data were analyzed by one-way ANOVA followed by Dunnett’s multiple comparisons test.
*p < 0.05, **p < 0.01, ***p < 0.001 versus control.

Legend: Chronic ASAE exposure did not induce impairment in basal physiological parameters associated with survival, locomotor performance, feeding behavior, or reproductive capacity in *C. elegans*. In contrast, ASAE exposure was associated with preservation of neuromuscular activity and reduction of oxidative stress-related biomarkers, supporting the absence of overt basal toxicity under the experimental conditions.

**Supplementary Table S4. Modulation of conserved stress-response pathways following ASAE exposure in transgenic *C. elegans strains*. Data are expressed as mean ± SEM.**

| Strain | Reporter / Pathway | Endpoint Evaluated | Control (% control) | ASAE 10 mg/mL | ASAE 25 mg/mL | ASAE 50 mg/mL |
| --- | --- | --- | --- | --- | --- | --- |
| TJ356 | DAF-16::GFP (FOXO pathway) | Nuclear localization (% worms) | 18 ± 3 | 29 ± 4* | 46 ± 5** | 63 ± 6*** |
| CF1553 | sod-3::GFP (oxidative stress response) | Fluorescence intensity (% control) | 100 ± 5 | 118 ± 6* | 151 ± 8** | 192 ± 10*** |
| GA800 | ctl-1/2/3-associated catalase response | Relative catalase activity (% control) | 100 ± 4 | 116 ± 5* | 142 ± 7** | 171 ± 8*** |
| CL2166 | gst-4::GFP (SKN-1/NRF2 pathway) | Fluorescence intensity (% control) | 100 ± 6 | 127 ± 7* | 166 ± 9** | 214 ± 11*** |

Data were analyzed using one-way ANOVA followed by Dunnett’s multiple comparisons test.
*p < 0.05, **p < 0.01, ***p < 0.001 versus control.

Legend: ASAE exposure promoted activation of conserved stress-response pathways associated with DAF-16/FOXO and SKN-1/NRF2 signaling in transgenic *C. elegans* strains. Increased DAF-16 nuclear localization and elevated sod-3, catalase-associated, and gst-4 responses support the involvement of antioxidant and cytoprotective mechanisms in the biological effects induced by ASAE.

**Supplementary Table S5. Baseline cholinergic activity and neuronal integrity following chronic ASAE exposure in *C. elegans* under non-stress conditions.** Data are expressed as mean ± SEM.

| Treatment | Relative AChE Activity (% control) | Pharyngeal Pumping (pumps/10 s) | LX929 Cholinergic Fluorescence (% control) |
| --- | --- | --- | --- |
| Control | 100 ± 5 | 14.8 ± 0.5 | 100 ± 4 |
| ASAE 10 mg/mL | 98 ± 4 | 14.6 ± 0.4 | 102 ± 5 |
| ASAE 25 mg/mL | 96 ± 5 | 14.9 ± 0.6 | 104 ± 4 |
| ASAE 50 mg/mL | 94 ± 4 | 15.1 ± 0.5 | 103 ± 5 |

Data were analyzed using one-way ANOVA followed by Dunnett’s multiple comparisons test. No statistically significant differences were observed between ASAE-treated groups and control animals under basal non-stress conditions (p > 0.05).

Legend: Baseline cholinergic function was evaluated in synchronized adult *C. elegans* following chronic ASAE exposure under non-stress conditions. Acetylcholinesterase (AChE) activity, pharyngeal pumping behavior, and cholinergic neuronal fluorescence in the LX929 strain were not significantly altered by ASAE treatment, supporting the absence of detectable basal cholinergic dysfunction or cholinergic neurodegeneration in the absence of chemical stressors.
